# Identifying inhibitory compounds in lignocellulosic biomass hydrolysates using an exometabolomics approach

**DOI:** 10.1186/1472-6750-14-22

**Published:** 2014-03-21

**Authors:** Ying Zha, Johan A Westerhuis, Bas Muilwijk, Karin M Overkamp, Bernadien M Nijmeijer, Leon Coulier, Age K Smilde, Peter J Punt

**Affiliations:** 1 TNO Microbiology & Systems Biology, Utrechtsweg 48, Zeist 3704 HE, The Netherlands; 2 Netherlands Metabolomics Centre (NMC), Einsteinweg 55, Leiden 2333 CC, The Netherlands; 3 Biosystems Data Analysis, Swammerdam Institute for Life Sciences, University of Amsterdam, Science Park 904, 1098 XH Amsterdam, The Netherlands; 4 TNO Triskelion BV, Utrechtseweg 48, Zeist 3700 AV, The Netherlands; 5 TNO Quality & Safety, Utrechtseweg 48, 3704 HE Zeist, The Netherlands

**Keywords:** Lignocellulosic biomass hydrolysate, Inhibitor, Metabolomics, Fermentation, EA-GC-MS, EC-GC-MS, (n)PLS model, Double cross validation

## Abstract

**Background:**

Inhibitors are formed that reduce the fermentation performance of fermenting yeast during the pretreatment process of lignocellulosic biomass. An exometabolomics approach was applied to systematically identify inhibitors in lignocellulosic biomass hydrolysates.

**Results:**

We studied the composition and fermentability of 24 different biomass hydrolysates. To create diversity, the 24 hydrolysates were prepared from six different biomass types, namely sugar cane bagasse, corn stover, wheat straw, barley straw, willow wood chips and oak sawdust, and with four different pretreatment methods, i.e. dilute acid, mild alkaline, alkaline/peracetic acid and concentrated acid. Their composition and that of fermentation samples generated with these hydrolysates were analyzed with two GC-MS methods. Either ethyl acetate extraction or ethyl chloroformate derivatization was used before conducting GC-MS to prevent sugars are overloaded in the chromatograms, which obscure the detection of less abundant compounds. Using multivariate PLS-2CV and nPLS-2CV data analysis models, potential inhibitors were identified through establishing relationship between fermentability and composition of the hydrolysates. These identified compounds were tested for their effects on the growth of the model yeast, *Saccharomyces. cerevisiae* CEN.PK 113-7D, confirming that the majority of the identified compounds were indeed inhibitors.

**Conclusion:**

Inhibitory compounds in lignocellulosic biomass hydrolysates were successfully identified using a non-targeted systematic approach: metabolomics. The identified inhibitors include both known ones, such as furfural, HMF and vanillin, and novel inhibitors, namely sorbic acid and phenylacetaldehyde.

## Background

Lignocellulosic biomass, like bagasse, wheat straw, and corn stover, is the 2^nd^ generation feedstock for biofuel production. Compared to fossil fuel, it is abundant, renewable and environmental friendly, while compared to 1^st^ generation feedstock, like corn, it does not compete with world food supply [1,2]. Lignocellulosic biomass is composed of cellulose, hemicellulose and lignin, of which cellulose is the homopolymer of glucose, while hemicellulose is a heteropolymer mainly composed of glucose and xylose [3,4]. A pretreatment step is required to break down the structure of lignocellulosic biomass and expose cellulose for hydrolysis [5,6]. The hydrolysis product, so-called biomass hydrolysate, is used as substrate for biofuel production through fermentation processes [7]. During most biomass pretreatment processes, harsh conditions, like high temperature and high pressure, were adopted. This causes sugars and lignin in biomass hydrolysates to degrade, forming products that possess inhibitory effects towards fermenting hosts, thus resulting in reduced growth and productivity [8-11].

**Figure 1:**
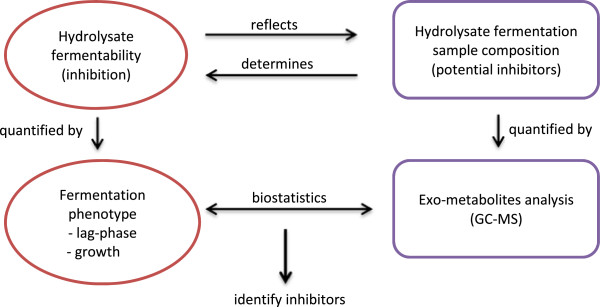
Graphic illustration of the concept of the exometabolomics approach.

Research has been conducted in other laboratories to identify compounds in biomass hydrolysates that cause inhibitory effects [12-14]. For these studies, it was found that inhibitors fall into three categories, weak acids (e.g. acetic acid), furans (e.g. furfural, HMF) and phenolic compounds (e.g. vanillin) [15-17]. A variety of experimental and analytical methods were used in these studies for identifying inhibitory compounds. A common feature of these studies was the approach that was used [18], namely, the selection of inhibitory compounds to test for their toxicity based on literature research without hydrolysate composition analysis followed by hydrolysate toxicity test towards fermenting microorganisms [11,19,20]. Besides the identified inhibitors, evidence was obtained showing that other compounds present in biomass hydrolysates also display inhibitory effects [21,22]. They were observed as unknown peaks in hydrolysate compositional analysis results, which reduced in size after detoxification [23]. In this study a non-targeted exometabolomics approach was applied to identify novel inhibitory compounds in biomass hydrolysates. Generally, metabolomics is one of the ‘omics’ tools that studies the performance of research objects by analyzing their overall compositions [24,25]. In this study, research objects are lignocellulosic biomass hydrolysates, which are used as fermentation media for bioethanol production. The performance of biomass hydrolysates as fermentation media vary due to the difference in their compositions, i.e. inhibitory compounds and their concentrations. By establishing the relationship between composition and yeast performance in different biomass hydrolysates statistically, compounds that possess inhibitory effect could be indicated in an unbiased way (Figure 1).

In metabolomics, the search for important metabolites responsible for a certain response, e.g. fermentability, is often performed with multivariate data analysis methods [26,27]. These multivariate methods are able to search for the interactions between metabolites that are responsible for the response that is modeled. Partial least squares (PLS) is a multivariate data analysis method that is commonly used in metabolomics to search for the important metabolites [28]. As an extension of the PLS method, also n-way PLS may be used when the data-set consists of a time series as in the case of our metabolomics experiments. Rigorous validation of these models is necessary as multivariate data analysis methods may lead to false positive correlations [29,30]. Therefore, we decided to use double cross validation (2CV) to obtain unbiased prediction errors for the (n)PLS models [31,32].

We report here the detailed procedure and the results of using an exometabolomics approach for identifying inhibitory compounds in lignocellulosic biomass hydrolysates. This includes the batch fermentability of 24 different biomass hydrolysates using baker’s yeast, *S. cerevisiae* CEN.PK113-7D, and the analysis results of the fermentation samples by two GC-MS methods; the statistical model building procedure for identifying potential inhibitory compounds, and the toxicity testing results of the identified potential inhibitors. The results of this study show that of the potential inhibitory compounds indicated by the statistical models, a large fraction indeed exhibited inhibitory effects on the growth of fermenting yeast. These compounds consist of both known inhibitors, such as furfural and HMF, and novel inhibitors.

## Results

### Biomass hydrolysates preparation

To successfully identify inhibitory compounds in biomass hydrolysates with statistical models, acquiring hydrolysates with diverse performance is of importance [18]. 24 different hydrolysates were prepared from six different biomass and by using four hydrolysate preparation methods to achieve this (see Section Biomass hydrolysate preparation and fermentation). Among the six biomass, wheat straw, barley straw and corn stover are agricultural wastes, bagasse is a sugar industry byproduct, and willow and oak are wood products. Each of the six biomass was pretreated with four different methods, which used 2% sulfuric acid, 72% sulfuric acid, lime, and peracetic acid, respectively. The resulting 24 hydrolysates were tested for their performance as fermentation media on a small scale (ml), showing that there was a significant diversity among these 24 hydrolysates [33]. These hydrolysates were prepared in larger quantity (l) for the exometabolomics study. A batch fermentation of 1 l working volume was carried out for each hydrolysate based on previously developed procedures (see Section Biomass hydrolysate preparation and fermentation and [11]).

### Defining phenotypes

Identical batch fermentations were carried out for each of the 24 different hydrolysates generated. The fermentability was monitored by measuring OD600 (refer to as ‘OD’ in the following text), glucose and ethanol concentrations of the samples taken with a fixed time interval. To quantify the fermentability of the hydrolysates, four phenotypes were defined, which are lag-phase, glucose consumption rate (Glu CR), ethanol production rate (EtOH PR) and ethanol yield (EtOH Y). The definition of these four phenotypes are given in Equation 1 to 4 (Eq1 to Eq4), and the measurement results of the fermentation samples were used to calculate these phenotypes.

(Eq1) $$\mathrm{lag}-\mathrm{phase}\left(h\right)=\mathrm{time}\;\mathrm{to}\;\mathrm{reach}\;2\%\left(\mathrm{ODmax}-\mathrm{ODmin}\right)$$

(Eq2) $$\begin{array}{l} \mathrm{Glu}\;\mathrm{CR}\;\left(g/1/h\right)=\mathrm{the}\;\mathrm{slope}\;\mathrm{of}\;\mathrm{the}\;\mathrm{linear}\;\mathrm{part}\;\mathrm{of}\;\mathrm{the}\\ \;\mathrm{glucose}\;\mathrm{consumption}\;\mathrm{curve}\;\\ \;\left(\mathrm{maximum}\;\mathrm{slope}\right) \end{array}$$

(Eq3) $$\begin{array}{l} \mathrm{EtOH}\;\mathrm{PR}\;\left(g/l/h\right)=\mathrm{the}\;\mathrm{slope}\;\mathrm{of}\;\mathrm{the}\;\mathrm{linear}\;\mathrm{part}\;\mathrm{of}\;\mathrm{the}\\ \;\mathrm{ethanol}\;\mathrm{production}\;\mathrm{curve}\\ \;\left(\mathrm{maximum}\;\mathrm{slope}\right) \end{array}$$

(Eq4) $$\begin{array}{l} \mathrm{EtOH}\;Y\left(g/g\right)=\mathrm{EtOHmax}/\mathrm{initial}\;\mathrm{glucose}\\ \;\mathrm{concentration} \end{array}$$

As shown in the phenotype definitions, lag-phase has time as unit (Eq1), which represents the duration before growth began. Since during lag-phase, the fermenting yeast adapt to the media composition for growth [34], a longer lag-phase indicates the presence of compounds that delay growth. Glucose consumption rate (Glu CR) is an indicator of the growth rate of the fermenting yeast, while ethanol production rate (EtOH PR) and ethanol yield (EtOH Y) describe the productivity of the fermenting yeast in a specific hydrolysate. For each of the 24 fermentations, these four phenotypes were calculated (Table 1). It should be mentioned that growth rate is one of the most commonly used phenotypes describing the performance of fermenting hosts. In this study, instead of using growth rate, we chose Glu CR to describe growth. This is because OD measurement results were not easily comparable due to sample characteristics, such as color differences among hydrolysates, and flocculation. To confirm that Glu CR is a good indicator of growth rate, we also measured growth rate based on OD development for some samples (Table 1). It can be seen that Glu CR has very similar trends as the OD based growth rate (Additional file 1). Since glucose measurements are more accurate than OD, we have decided to use Glu CR as an indicator of growth rate.

**Table 1:** Fermentability of the 24 biomass hydrolysates, expressed as the calculation results of the defined phenotypes

**Hydrolysate**	**Glucose**^**1 **^**(g/l)**	**Ethanol**^**2 **^**(g/l)**	**Lag-phase**^**3 **^**(h)**	**Glu CR**^**4 **^**(g/l/h)**	**EtOH PR**^**5 **^**(g/l/h)**	**EtOH Y**^**6 **^**(g/g)**	**Growth rate based on OD**^**7**^
**Bag-CA**	**67.39**	**20.61**	**7.5**	**1.42**	**0.44**	**0.306**	**2.42**
**Bag-DA**	**63.33**	**24.20**	**6.0**	**3.64**	**1.52**	**0.382**	**4.61**
**Bag-MA**	**58.82**	**22.71**	**2.0**	**3.84**	**1.58**	**0.386**	**5.73**
Bag-PAA	52.48	19.87	3.0	2.52	0.71	0.379	2.76
**BS-CA**	**67.45**	**30.92**	**7.5**	**4.57**	**1.73**	**0.458**	**7.39**
**BS-DA**	**49.87**	**20.95**	**6.5**	**3.63**	**1.42**	**0.420**	**5.66**
BS-MA	42.56	18.40	6.0	3.05	1.41	0.432	*
**BS-PAA**	**53.50**	**22.22**	**3.0**	**2.96**	**1.03**	**0.415**	**5.12**
**CS-CA**	**65.63**	**26.62**	**10.5**	**3.21**	**1.32**	**0.406**	**4.73**
**CS-DA**	**42.80**	**18.74**	**5.5**	**3.43**	**1.49**	**0.438**	**6.98**
**CS-MA**	**32.83**	**15.85**	**6.5**	**3.35**	**1.32**	**0.483**	**7.92**
CS-PAA	50.29	20.84	3.5	2.38	1.03	0.414	4.53
**Oak-CA**	**66.72**	**12.06**	**1.5**	**0.80**	**0.29**	**0.181**	**2.1**
**Oak-DA**	**38.22**	**15.27**	**5.0**	**2.41**	**0.98**	**0.400**	**5.37**
**Oak-MA**	**44.35**	**19.49**	**2.5**	**3.43**	**1.55**	**0.439**	**7.52**
**Oak-PAA**	**60.80**	**25.97**	**3.0**	**2.73**	**1.12**	**0.427**	**3.75**
Willow-CA	31.58	13.60	1.0	4.26	1.10	0.431	14.04
**Willow-DA**	**45.15**	**17.68**	**7.5**	**2.74**	**1.14**	**0.392**	**5.72**
Willow-MA	23.50	10.76	4.5	2.68	1.29	0.458	*
**Willow-PAA**	**51.30**	**22.81**	**5.5**	**2.45**	**1.05**	**0.445**	**5.03**
**WS-CA**	**60.54**	**24.71**	**9.0**	**4.63**	**1.87**	**0.408**	**7.6**
**WS-DA**	**58.29**	**24.83**	**4.5**	**3.47**	**1.64**	**0.426**	**6.05**
**WS-MA**	**32.12**	**13.95**	**6.5**	**4.01**	**1.92**	**0.434**	**11.37**
WS-PAA	51.94	21.61	3.5	3.03	1.27	0.416	5.48

^1^Glucose concentration of the 24 hydrolysates; ^2^final ethanol concentration; ^3^(Eq1); ^4^glucose consumption rate (Eq2); ^5^ethanol production rate (Eq3); ^6^ethanol yield (Eq4); ^7^the slope of the linear part of the OD% curve. *OD measurement was not possible due to flocculation; **bold**: fermentations that are selected for sample compositional analysis.

As shown in Table 1, all 24 hydrolysates had different glucose concentrations, indicating that biomass type as well as pretreatment method influenced the biomass hydrolysis efficiency. In general, mild alkaline (MA) pretreated biomass resulted in relatively low glucose concentration, while concentrated acid (CA) lead to higher hydrolysis efficiency [33]. However, based on our previous results, glucose concentration in the range observed in Table 1 had no influence on fermentation performance (results not shown).

The performance of the 24 hydrolysates varied significantly as fermentation media, which was consistent with the screening experiments on milliliter scale [33]. As far as lag-phase is considered, hydrolysates like Oak-CA and Willow-CA supported growth almost immediately after inoculation, while the fermenting yeast needed an adaptation period of as long as 10 hours in CS-CA and WS-CA hydrolysates. The Glu CR of the 24 hydrolysates ranged from 0.80 (Oak-CA) to 4.63 (WS-CA), which was comparable to that of EtOH PR (Additional file 2). This resulted in very similar ethanol yield among the hydrolysates, around 0.4 g/g (Table 1), which was also the ethanol yield of *S.cerevisiae* CEN.PK113-7D in mineral medium with 20 g/l glucose [35]. This observation suggested that under anaerobic conditions, the effect of inhibitory compounds in hydrolysates had little effect on the ethanol yield of the fermenting yeast. Therefore, this phenotype was not used in building statistical models for the purpose of identifying hydrolysate inhibitors.

Some had similar performance in terms of the calculated phenotypes among the 24 hydrolysate fermentations (Additional file 2). Since the statistical models to be used for analyzing the relationship between fermentability and sample composition were based on linear regression, it is important to reduce overrepresentation of certain phenotype classes. In addition, it is also beneficial to minimize the amount of samples for exometabolomics analysis. Therefore, from the 24 fermentations, 16 were selected based on the variations in their phenotypes, biomass type and pretreatment method. The selected 16 hydrolysates contain all six biomass types and all four biomass pretreatment methods (Table 1), and the fermentability of these selected hydrolysates show a more or less even spread of the fermentation phenotypes (Additional file 2).

### Hydrolysate fermentation sample analysis

After quantifying the performance of the hydrolysate fermentations with the four phenotypes, cell free time-point samples of the 16 selected fermentations were analyzed for their overall compositions. These samples were chosen based on the criteria that they should uniquely represent the whole fermentation process. The five fermentation time-point samples are listed in Table 2, which selection was based on the three fermentation phases, namely lag-phase, growth-phase and stationary-phase. The division of the three fermentation phases was consistent with the definition of the phenotypes, i.e. the end of lag-phase is when OD reaches 2% of the maximum OD, the end of growth-phase is when glucose consumption is completed, and the duration of stationary-phase is fixed at 10 hours after the end of growth phase. In this way, a total of 80 samples from 16 hydrolysate fermentations were selected for compositional analysis. Overall fermentation durations were within a 20 to 70 hours timeframe.

**Table 2:** The five fermentation time samples for compositional analysis with the two GC-MS methods

t1	Beginning of fermentation	Immediately after inoculation
t2	End of lag-phase	Time needed to reach 2% (ODmax-ODmin)
t3	Growth mid-point	Time needed to consume half of the initial glucose
t4	Growth end point	Time needed to consume all glucose
t5	Stationary phase	10 hours after growth end point

The focus of the compositional analysis was to identify potential inhibitory compounds in hydrolysate samples, which are believed to be mainly non-sugar compounds, such as weak acids, furans and phenols [8,9,15]. GC-MS was chosen as the analytical tool, as the method is capable of detecting a wide range of these compounds, including many unknowns [20,36]. A crucial point in analyzing hydrolysate samples with GC-MS was to remove sugars, which are present in large quantities in the samples and severely interfere with the detection of non-sugar compounds [13,37]. For this purpose, two sample work-up methods were developed, using ethyl acetate extraction orethyl chloroformate derivatization.

Ethyl acetate extraction GC-MS (EA-GC-MS) was previously described by Heer et al. [13]. The method uses ethyl acetate (EA) as solvent, in which compounds that are apolar, e.g. with aromatic rings, are dissolved, while polar compounds, like sugars, remain in the water phase. In current study, the hydrolysate samples were extracted twice with EA to allow adequate recovery of the extracts. After removing EA phase from the water phase, it was dried through evaporation, thus concentrated and ready for analysis with GC-MS.

Only compounds dissolvable in EA were analyzed due to the nature of this sample work-up method, and since EA was removed through evaporation, volatile compounds were partially lost. It was found that sample pH influences the extraction, when pH was raised to above 6.0, significant decrease of recovery was observed with multiple aromatic standards. Therefore, all hydrolysate samples were extracted with EA at pH 5.0. In doing so, the recovery of aromatic compounds was satisfying, above 90%, while the recovery of furans was rather low and inconsistent due to evaporation. So the analysis results of aromatic compounds were considered more reliable than furans.

Ethyl chloroformate derivatization GC-MS (EC-GC-MS) was developed in our lab to complement the EA-GC-MS method. Ethyl chloroformate (EC) was used to convert acids to their ethyl ester form, thus compounds like carboxylic acids, amino acids, aromatic compounds and furans could be detected by MS. EC-GC-MS therefore has a larger coverage of compounds compared to EA-GC-MS, and is easier to operate. But due to the diverse reactivity of compounds with EC, it is possible that compounds present with high concentration could only be detected with low signal. The involvement of a derivatization step could also cause a single compound to have more than one derivatization product, which complicates the characterization of the compound. EC-GC-MS method not only complemented EA-GC-MS by detecting small carboxylic acids and furans, but also overlapped with EA-GC-MS by detecting aromatic compounds. As far as aromatic compounds are concerned, it seems that the results of EA-GC-MS were more reliable due to the reactivity diversity issue occurring in EC-GC-MS.

After analyzing all 80 samples with both methods, a ‘compound list’ was generated for each method by listing all peaks clearly visible in the chromatograms. Identities were assigned to some of the peaks by comparing the mass spectra of these peaks with the existing GC-MS compound library in our lab. With the EA-GC-MS method, in total 129 peaks were detected, among which 44 were identified; while with EC-GC-MS, there were 114 detected peaks, of which 56 were identified. The majority of the compounds detected by EC-GC-MS method were acids, including carboxylic acids, such as levulinic acid and succinic acid, phenolic acids, like phenylacetic acid and syringic acid, and 18 amino acids (Additional file 3). EA-GC-MS mainly detected phenolic compounds, containing phenolic aldehydes, alcohols and acids (Additional file 3).

Pseudo-quantities were assigned to all detected peaks from both identified compounds and unknowns by integrating their peak areas. Internal standards were measured in both blank and hydrolysate sample to correct for sample matrix effect. The peak area difference between blank and hydrolysate sample of the internal standards was calculated as a correction-factor, and was used to correct all the integrated peak areas of the same hydrolysate type. Thus, compound lists based on corrected peak areas were formed for both analytical methods.

### Statistical model building

To identify inhibitory compounds in biomass hydrolysates, relationship between hydrolysate fermentability and fermentation sample composition was studied by building statistical models. The models used in this study were partial least square (PLS) and n-way PLS (nPLS), validated by conducting double cross validation (2CV), which was done by leave-one-out in the inner and outer loop [31,38]. The purpose of both models was to point to compounds that are most responsible for a certain fermentation phenotype. This was done by predicting the phenotypes using the data-sets formed through analyzing fermentation samples with the two GC-MS methods (see Section Hydrolysate fermentation sample analysis and Additional file 3).

#### Lag-phase

Lag-phase is the period before growth takes place in a fermentation process (Eq1), it is mainly influenced by the initial media composition. During lag-phase, fermenting yeast adapts to hydrolysate through adjusting its composition by either degrading or converting compounds [39,40]. Therefore, it is reasonable to describe lag-phase by comparing the composition difference between sample t1 and t2 (model 1, 2 and 3 in Table 3). In addition, the composition of sample t1 and t2 represents the beginning and the end point of the lag-phase (Table 2), which was also used to build models for predicting lag-phase (models 4, 5 and 6 in Table 3).

**Table 3:** Data-sets used for building PLS-2CV models

Lag-phase model 1	$\mathrm{auto}\left(\sqrt{EA_{t2}}-\sqrt{EA_{t1}}\right)$
Lag-phase model 2	$\mathrm{auto}\left(\sqrt{EC_{t2}}-\sqrt{EC_{t1}}\right)$
Lag-phase model 3	$\mathrm{auto}\left(\left(\sqrt{EA_{t2}}-\sqrt{EA_{t1}}\right):\left(\sqrt{EC_{t2}}-\sqrt{EC_{t1}}\right)\right)$
Lag-phase model 4	$\mathrm{auto}\left(\sqrt{EA_{t1}:EA_{t2}}\right)$
Lag-phase model 5	$\mathrm{auto}\left(\sqrt{EC_{t1}:EC_{t2}}\right)$
Lag-phase model 6	autoEA:t1EAt2:ECt1:ECt2
Glu CR and EtOH PR	$\mathrm{auto}\left(\sqrt{EA_{\mathrm{tx}}}\right)\;\mathrm{or}\;\mathrm{auto}\left(\sqrt{EC_{\mathrm{tx}}}\right)$

The two data preprocessing methods were symbolized by ‘√’ (square-root), and ‘*auto*’ (autoscaling); ‘:’ indicates that the corresponding data-sets were combined. EA_tx_: EA-GC-MS data-set of time sample tx, EC_tx_: EC-GC-MS data-set of time sample tx (tx represents fermentation samples taken at different time-points; x: 1-5).

In total, six models were constructed for lag-phase (Table 3), of which the data-sets of EA-GC-MS and EC-GC-MS methods were used both separately and combined. This is because the effect of fusing these two data-sets was unknown. The prediction results of the six models are shown in Table 4. It can be seen that among the six models, only ‘model 2’ and ‘model 5’ had a Q^2^ value above 0.5, indicating that these two models are valid and could be used to predict lag-phase (refer to PLS-2CV models in Method section for the definition of the Q^2^ value). As shown in Table 3 that the inputs of both ‘model 2’ and ‘model 5’ were from the EC-GC-MS data-set, suggesting that the compounds detected by EC-GC-MS method had more influence on lag-phase compared to those measured with EA-GC-MS.

**Table 4:** **Lag-phase prediction results and Q**^**2 **^**values of the PLS-2CV models shown in Table**3

**PLS-2CV**	**Lag-phase**	**Prediction**					
		**Model 1**	**Model 2**	**Model 3**	**Model 4**	**Model 5**	**Model 6**
Bag-CA	7.5	6.13	**9.28**	7.43	5.79	**6.18**	**4.96**
Bag-DA	6.0	3.36	**5.24**	4.61	6.79	**4.95**	**5.53**
Bag-MA	2.0	5.02	**3.55**	4.27	4.21	**3.08**	**2.90**
BS-CA	7.5	7.14	**7.67**	7.45	7.42	**9.28**	**8.44**
BS-DA	6.5	8.99	**5.29**	8.23	9.49	**6.57**	**7.71**
BS-PAA	3.0	3.61	**4.12**	3.82	3.86	**4.67**	**3.86**
CS-CA	10.5	11.71	**9.44**	10.91	10.32	**10.61**	**11.33**
CS-MA	6.5	−6.10	**6.28**	−1.36	4.40	**6.66**	**6.25**
Oak-CA	1.5	4.25	**4.48**	4.43	6.27	**6.02**	**6.19**
Oak-DA	5.0	4.42	**5.70**	4.74	6.16	**5.94**	**5.81**
Oak-MA	2.5	3.81	**4.00**	3.99	−3.28	**2.00**	**−0.54**
Oak-PAA	3.0	5.39	**4.38**	5.05	3.47	**2.99**	**2.55**
Willow-DA	7.5	6.83	**8.95**	7.61	5.19	**6.70**	**6.68**
Willow-PAA	5.5	3.61	**6.08**	4.36	7.12	**2.19**	**4.51**
WS-CA	9.0	7.93	**8.17**	8.00	6.95	**6.86**	**6.18**
WS-MA	6.5	6.58	**2.01**	3.97	4.38	**7.95**	**7.59**
Q^2^		−1.01	**0.54**	0.06	0.05	**0.51**	**0.47**

**Bold**: models selected for analyzing the SR of the peaks in the data-sets.

Models which use EA-GC-MS data-set failed to predict lag-phase properly (‘model 1’ and ‘model 4’ in Table 4), but when combined with EC-GC-MS data-set, the prediction improved, resulting in models with Q^2^ value of 0.06 and 0.47 (‘model 3’ and ‘model 6’, respectively, in Table 4). As the Q^2^ value of ‘model 6’ is very close to 0.5, this model was still selected, together with models 2 and 5, to calculate the selectivity ratios (SR) assigned to each peaks in these data-sets.

For each detected peaks from EC-GC-MS data-set, the SR values of the three models in bold in Table 4 were summed, and ranked based on their SR-sum values; while for each detected peaks in EA-GC-MS data-set, the SR value of ‘model 6’ were ranked (refer to Section PLS-2CV models in Method section for the definition of the SR value). The top 40 peaks with the highest SR-sum values, 20 from EC-GC-MS data-set and 20 from EA-GC-MS data-set, were considered as the main contributors in predicting lag-phase. Among these 40 peaks, the ones with identity were tested for their effects on the fermenting yeast (Section Potential inhibitory compound testing, Table 5). The detailed ranking procedure of lag-phase model SR is shown in Additional file 4.

**Table 5:** Inhibitory effects of the compounds identified by lag-phase, Glu CR and EtOH PR models, tested using Bioscreen C Analyzer

**Reference medium (MM with 20 g/l glucose)**					**LP**	**GR**	**OD**			
					**7 h**	**0.105**	**1.2**			
**Compounds**	**Structure**	**0.2 g/l**			**0.5 g/l**			**1.0 g/l**		
		**LP**	**GR**	**OD**	**LP**	**GR**	**OD**	**LP**	**GR**	**OD**
**Compounds identified by all 3 phenotype models**										
Furfural		10 h	---	---	15 h	---	---	30 h	No growth	
HMF		---	---	---	---	---	---	---	< 20%	---
**Sorbic acid**		---	< 60%	< 80%	---	< 60%	< 80%	No growth		
**Syringaldehyde**		---	---	---	---	---	---	---	< 60%	< 80%
**Suberic acid**								*3 h*	*110%*	*110%*
**Compounds identified by lag-phase model**										
**Benzoic acid**		---	< 60%	< 80%	---	< 40%	< 80%	---	< 40%	< 60%
**Compounds identified by Glu CR and EtOH PR models**										
*Phenylacet aldehyde*		20 h	< 80%	---	No growth			No growth		
Vanillin		---	---	---	11 h	< 80%	---	30 h	No growth	
*4-hydroxybenzaldehyde*		---	---	---	*9.5 h*	< 80%	---	*11 h*	< 60%	< 80%
Dihydroxy benzene		---	---	---	---	---	---	---	< 80%	---
**Conifer aldehyde**		---	---	---	23 h	---	---	No growth		
**Compounds identified by Glu CR model**										
**Ferulic acid**		---	---	---	---	---	---	---	*< 40%*	*< 80%*
*Benzaldehyde*		---	---	---	9.5 h	< 80%	---	13.5 h	< 80%	< 80%
**Compounds identified by EtOH PR model**										
**Salicylic acid**		---	*< 80%*	*< 80%*	---	*< 60%*	*< 80%*	---	*< 60%*	*< 60%*
No effect										
2-furoic acid		Pantolactone				Levulinic acid				
Furfuryl alcohol		2-furanmethanol acetate				HMF alcohol				
*P*-coumaric acid		Homovanillic acid				HMF acid				
3-phenyllactic acid		4-hydroxybenzoic acid				Vanillic acid				
Not tested										
*4-Hydroxyphenylaldehyde*		Phloretic acid				5-HMF methyl keton				

LP: lag-phase: time needed to reach 2% (ODmax-ODmin) (h), GR: growth rate: the slope of the linear part of the OD curve (OD/h), OD: final OD. Values with% are relative growth rate and final OD compared to that in reference medium. ‘---’: no effect compared to reference medium.

The compounds indicated in ‘*italic*’ were originally identified by their (converted and less toxic) alcohol forms; the compounds indicated in ‘bold’ were saturated when 1 g/l solutions were prepared at the fermentation temperature, in these cases, besides the saturated solution, a 2- and 5- fold dilution was used, represented in the 0.5 and 0.2 g/l columns, respectively; the ‘italic effects’ were NOT observed when tested in YPD medium.

#### Glu CR and EtOH PR

Unlike lag-phase, samples taken at all five time-points influence Glu CR and EtOH PR according to their definitions (Eq2 and Eq3). These phenotypes were modeled by the data-sets of the five fermentation samples both individually, using the PLS-2CV model, and collectively, using the nPLS-2CV model.

PLS-2CV modeled Glu CR and EtOH PR with the data-sets of individual fermentation samples, which reveals the influence of these single time points on these two phenotypes. The modeling results show that EC-GC-MS data-sets failed to predict Glu CR and EtOH PR, as the resulting Q^2^ values were all negative (data not shown). On the contrary, the EA-GC-MS data-sets of sample t3, t4 and t5 successfully modeled the two phenotypes, as shown in Table 6, the resulting Q^2^ values were above 0.5. This suggests that, different from lag-phase, Glu CR and EtOH PR were relating to the compounds detected with EA-GC-MS method. Moreover, the prediction became meaningful only after time point t2 (Table 6, Q^2^ > 0), indicating that Glu CR and EtOH PR were not affected by the initial hydrolysate composition, but influenced by the composition after lag-phase and during growth. This confirms that the data-sets of time point t1 and t2 possess a different structure compared to the other three time points. This structure contains information that could properly describe lag-phase (Table 4), which ends after time point t2, but failed to predict Glu CR and EtOH PR, which describe a different phase of the fermentation process.

**Table 6:** **Glu CR and EtOH PR prediction results and Q**^**2 **^**values of the PLS-2CV models**

**PLS-2CV**	**Glu CR**	**Prediction**					**EtOH PR**	**Prediction**				
		t1	t2	**t3**	**t4**	**t5**		t1	t2	**t3**	**t4**	**t5**
Bag-CA	1.42	2.86	2.30	**2.08**	**1.77**	**1.08**	0.44	1.04	0.92	**0.81**	**0.81**	**0.47**
Bag-DA	3.64	2.62	3.07	**3.00**	**3.01**	**3.56**	1.52	0.93	1.15	**1.18**	**1.18**	**1.39**
Bag-MA	3.84	3.12	3.28	**3.64**	**3.45**	**3.22**	1.58	1.38	1.13	**1.51**	**1.51**	**1.40**
BS-CA	4.57	2.38	3.94	**4.15**	**3.71**	**3.88**	1.73	0.91	1.61	**1.70**	**1.70**	**1.61**
BS-DA	3.63	4.60	3.99	**3.73**	**3.93**	**3.45**	1.42	1.99	1.72	**1.57**	**1.57**	**1.47**
BS-PAA	2.96	2.39	2.80	**2.73**	**2.81**	**2.65**	1.03	1.04	1.20	**1.13**	**1.13**	**1.13**
CS-CA	3.21	2.98	3.51	**3.71**	**3.24**	**3.51**	1.32	1.12	1.31	**1.44**	**1.44**	**1.35**
CS-MA	3.35	4.21	4.52	**4.00**	**4.23**	**4.31**	1.32	1.85	2.03	**1.81**	**1.81**	**1.89**
Oak-CA	0.80	2.88	2.76	**1.77**	**1.94**	**2.00**	0.29	1.03	1.09	**0.57**	**0.57**	**0.66**
Oak-DA	2.41	2.54	3.04	**2.63**	**1.83**	**2.35**	0.98	1.23	1.46	**1.22**	**1.22**	**1.22**
Oak-MA	3.43	2.67	2.70	**3.03**	**2.98**	**3.12**	1.55	1.01	1.04	**1.20**	**1.20**	**1.25**
Oak-PAA	2.73	2.95	3.01	**2.63**	**2.59**	**2.66**	1.12	1.13	1.18	**0.99**	**0.99**	**1.00**
Willow-DA	2.74	2.78	2.61	**2.89**	**3.31**	**2.83**	1.14	1.15	0.99	**1.16**	**1.16**	**1.13**
Willow-PAA	2.45	3.70	3.06	**3.21**	**3.48**	**3.39**	1.05	1.46	1.23	**1.32**	**1.32**	**1.35**
WS-CA	4.63	3.43	3.74	**3.49**	**3.67**	**3.49**	1.87	0.93	1.42	**1.33**	**1.33**	**1.32**
WS-MA	4.01	4.10	3.29	**3.47**	**3.43**	**3.37**	1.92	1.63	1.29	**1.41**	**1.41**	**1.37**
Q^2^		−0.161	0.374	**0.676**	**0.574**	**0.607**		−0.315	0.044	**0.539**	**0.500**	**0.555**

**Bold**: models selected for analyzing the SR of the peaks in the EA-GC-MS data-sets.

To include the effect of hydrolysate composition change during the fermentation process on Glu CR and EtOH PR, the five time-point samples were also analyzed collectively, with the nPLS-2CV model. Consistent with the PLS-2CV models, the prediction was only valid with EA-GC-MS data-set (Table 7). Since it was known from PLS-2CV models that data-set of sample t1 gave negative Q^2^ values (Table 6), nPLS-2CV models were also built with the data-set of sample t2 to t5. As shown in Table 7, the predictions of Glu CR and EtOH PR were improved when sample t1 was excluded from the data-set, indicating that the input of sample t1 data-set was negative.

**Table 7:** **Glu CR and EtOH PR prediction results and Q**^**2 **^**values of the nPLS-2CV models**

**nPLS-2CV**	**Glu CR**	**Prediction**		**EtOH PR**	**Prediction**	
		**t1 - t5**	**t2 - t5**		**t1 - t5**	**t2 - t5**
Bag-CA	1.42	1.55	**1.41**	0.44	1.17	**0.59**
Bag-DA	3.64	2.87	**3.07**	1.52	1.05	**1.16**
Bag-MA	3.84	3.16	**3.25**	1.58	1.27	**1.32**
BS-CA	4.57	3.76	**3.67**	1.73	1.53	**1.51**
BS-DA	3.63	4.02	**3.91**	1.42	1.69	**1.64**
BS-PAA	2.96	2.79	**2.88**	1.03	1.20	**1.23**
CS-CA	3.21	3.49	**3.54**	1.32	1.35	**1.36**
CS-MA	3.35	4.40	**4.37**	1.32	2.00	**2.00**
Oak-CA	0.80	1.96	**1.86**	0.29	0.65	**0.62**
Oak-DA	2.41	2.28	**2.35**	0.98	1.17	**1.08**
Oak-MA	3.43	2.71	**2.86**	1.55	1.05	**1.11**
Oak-PAA	2.73	2.81	**2.78**	1.12	1.08	**1.07**
Willow-DA	2.74	2.83	**2.90**	1.14	1.16	**1.18**
Willow-PAA	2.45	3.68	**3.55**	1.05	1.49	**1.42**
WS-CA	4.63	3.70	**3.64**	1.87	1.40	**1.39**
WS-MA	4.01	3.58	**3.46**	1.92	1.41	**1.37**
Q^2^		0.526	**0.580**		0.182	**0.419**

**Bold**: models selected for analyzing the SR of the peaks in the EA-GC-MS data-sets.

Thus, for Glu CR and EtOH PR, three PLS-2CV models and a nPLS-2CV model were selected respectively for analyzing the contributions of the peaks in EA-GC-MS data-set to model predictions (models with ‘Bold’ in Table 6 and Table 7). With PLS-2CV models, similar to lag-phase, SR of the peaks were summed and ranked. The top 40 peaks with the highest SR values were considered as the main contributors of PLS-2CV models of either Glu CR or EtOH PR. While with nPLS-2CV models, the regression coefficient (‘reg’) values were used for ranking. The top 40 peaks with the highest absolute ‘reg’, 20 with positive values and 20 with negative values, were considered as the main contributor of nPLS-2CV model of either Glu CR or EtOH PR. Among the selected peaks, the ones with identity were tested for their effects on the fermenting yeast (Section Potential inhibitory compound testing, Table 5). The detailed ranking and selection procedure of the testing compounds are shown in Additional file 5. Interestingly, more than 80% of the compounds identified by Glu CR and EtOH PR models are identical. This indicates, from a statistical point of view, the correlation between Glu CR and EtOH PR.

### Potential inhibitory compound testing

Two groups of potential inhibitory compounds were identified, one from lag-phase models and the other from Glu CR and EtOH PR models, through constructing statistical models and analyzing the compounds that contribute the most to the models with valid phenotype predictions. Growth tests using Bioscreen C Analyzer were conducted in mineral medium (MM) with 20 g/l glucose to study the effect of these compounds on the fermenting yeast, *S. cerevisiae* CEN.PK113-7D. The potential inhibitory compounds were added individually with the following three concentrations, 0.2, 0.5 and 1.0 g/l, respectively.

It should be noted that these testing concentrations could be much higher compared to that in actual biomass hydrolysates, i.e. less than 0.1 g/l [11,14,41,42]. The toxicity threshold of a specific compound can be much lower compared to that was tested in synthetic medium due to the synergistic effects present in biomass hydrolysates. Although the testing concentrations were higher compared to that in biomass hydrolysates, the testing results still provide meaningful information.

The first group of compounds shown in Table 5 were identified by all three phenotype models, among which, furfural resulted in longer lag-phase at all three concentrations tested, while sorbic acid and syringaldehyde reduced growth rate. Suberic acid exhibited positive effect towards the fermenting yeast, mainly through shortening lag-phase. Since this phenomenon was only observed in MM, but not in YPD, which a much richer medium compared to MM, we reason that the acid was probably used as a nutrient by the yeast. HMF, though known as an important inhibitor in biomass hydrolysates [43-45], only exhibited inhibitory effect at 1.0 g/l on the growth rate of the fermenting yeast (Table 5). However, HMF seems to prolong lag-phase when tested together with other compounds identified by the lag-phase models. It can be seen that HMF triggered synergistic effect with levulinic acid, 2-furoic acid and pantoyllacton, respectively, at 0.5 g/l (Table 8). This may be the reason why HMF was identified, though little effect was observed when tested individually.

**Table 8:** Compounds that caused synergistic effect with furfural or HMF at 0.5 g/l, tested using Bioscreen C Analyzer

**Reference medium**		**LP**	**GR**	**OD**			
**(MM with 20 g/l glucose)**		**7 h**	**0.105**	**1.2**			
	**Added compound**	**Added compound only**			**Added compound + furfural or HMF**		
		**LP**	**GR**	**OD**	**LP**	**GR**	**OD**
Furfural	HMF	---	---	---	**19 h**	**< 80%**	---
0.5 g/l	HMF acid	---	---	---	16.5 h	---	---
15 h	Salicylic acid	---	< 60%	< 80%	15 h	**< 40%**	< 60%
90%	Vanillin	11 h	< 80%	---	17 h	< 80%	---
100%	Syringaldehyde	---	---	---	16.5 h	**< 80%**	---
	Levulinic acid	---	---	---	9 h	**< 80%**	---
HMF	2-furoic acid	---	---	---	8.5 h	**< 80%**	---
0.5 g/l	Pantoyllacton	---	---	---	9 h	**< 80%**	---
7 h	Salicylic acid	---	< 60%	< 80%	8.5 h	**< 40%**	< 60%
100%	Vanillin	11 h	< 80%	---	11 h	**< 60%**	---
100%	Syringaldehyde	---	---	---	**11 h**	---	---

LP: lag-phase: time needed to reach 2% (ODmax-ODmin) (h), GR: growth rate: the slope of the linear part of the OD curve (OD/h), OD: final OD. Values with% are relative growth rate and final OD compared to that in reference medium. ‘---’: no effect compared to reference medium; the ‘bold effects’ were relatively significant.

Furfural was identified as a key toxin in biomass hydrolysates [13,46], and consistent with the current study, its main inhibitory effect was elongating lag-phase [47-49]. It was reported earlier that furfural as well as HMF are converted to their alcohol form (furfuryl alcohol and HMF alcohol) and eventually acid form (furoic acid and HMF acid) by the fermenting yeast due to detoxification [39,40]. This was also observed in this study. During lag-phase, the concentration of furfural and HMF decreased, while their alcohols and acids were formed. Since the concentration of furfuryl alcohol and 2-furoic acid is showing an opposite pattern compared to furfural, and HMF alcohol to HMF, as could be expected, these compounds were also identified by analyzing the lag-phase models (Table 5).

The potential inhibitors identified by Glu CR and EtOH PR models were mainly phenolic compounds (Table 5). It is known from previous research that the toxic form of a phenolic compound is often the aldehyde, which is converted to its alcohol during the fermentation process due to detoxification [20,22,50]. Therefore, possible conversion of the phenolic alcohols identified by the models was checked. For those phenolic alcohol compounds with increased concentrations during the fermentation process, the aldehyde forms were used in the growth tests, assuming that the alcohols were the conversion products. These phenolic aldehydes are marked in italic in Table 5. In agreement with former studies, the compounds exhibited inhibitory effects were mostly aldehydes and acids (Table 5). The major inhibitory effects were reduced growth rate and lower final OD. Phenylacetaldehyde, vanillin and conifer aldehyde caused growth deficiency at 1.0 g/l (0.5 g/l for phenylacetaldehyde, Table 5).

Besides the compounds listed in Table 5, another group of compounds identified by the models was the amino acids, of which concentrations decreased during the fermentation process. This provides the possibility that the depletion of amino acids in hydrolysates worsened the fermentation performance of the fermenting yeast. However, as growth of the fermenting yeast in hydrolysates was not improved when amino acids were added (data not shown), this was apparently not the case. Another explanation would be that the presence of amino acids and possibly other nutrients compensates the inhibitory effects of the inhibitors. This assumption was verified by comparing the inhibitory effects of the compounds listed in Table 5 in MM and YPD medium, which contains abundant peptides and nutrients compared to MM. The inhibitory effects of all the tested compounds alleviated in YPD medium, particularly, the effects in italic in Table 5 were absent in YPD. This observation indicates that the toxicity of inhibitors was culture medium dependent, suggesting that the fermentability of biomass hydrolysates could be improved by adding extra nutrients like yeast extract [51].

Furfural and HMF are the two most studied inhibitors in biomass hydrolysates, in terms of their inhibitory effects as well as their conversion pathways [13,40,45]. However, research on the synergistic effects of these two compounds with other potential inhibitors in hydrolysates was seldom tackled. In this study, the combined inhibitory effects of furfural or HMF with one other potential inhibitory compound were tested using Bioscreen C Analyzer at 0.5 g/l in MM with 20 g/l glucose, and the compounds demonstrated synergistic effect with either furfural or HMF are listed in Table 8. It can be seen that HMF caused a notable synergistic effect with levulinic acid, 2-furoic acid, pantoyllacton and syringaldehyde, respectively. These compounds showed no inhibitory effect individually at 0.5 g/l, but when added together with HMF, they extended lag-phase as well as reduced growth rate (Table 8). Compared to HMF, furfural combined with the selected compounds caused minor negative synergism, since no significant lag-phase increase or growth rate reduction was observed when an extra compound was added (Table 8).

## Discussion

Lignocellulosic biomass is a natural resource that has the potential to become the major feedstock for biofuel production [52,53]. A metabolomics approach was adopted in this study to identify inhibitory compounds in biomass hydrolysates. Compared to targeted methods, no compound pre-selection was made with the metabolomics approach, so that the inhibitor identification was not influenced by prior knowledge [18,26]. The study results show that the metabolomics approach successfully identified compounds that influence the growth of the fermenting yeast, *S. cerevisiae* CEN.PK 113-7D. Some compounds prolonged lag-phase, like furfural and vanillin, while others reduced growth rates, such as HMF and benzaldehyde. Interestingly, without pre-selection, compounds that were previously known as inhibitors in biomass hydrolysates were identified in this study. This confirms that metabolomics is a relevant approach in studying the composition and identifying inhibitors of lignocellulosic biomass hydrolysates.

As the analysis targets were potential inhibitory compounds in biomass hydrolysates, which are weak acids, furans and phenolic compounds [15-17], GC-MS was chosen as the analytical tool [18]. Either ethyl acetate (EA) extraction or ethyl chloroformate (EC) derivatization was conducted prior to sample analysis to prevent sugars from being in the final extracted hydrolysate samples. Due to the property difference of these two sample preparation methods, their target compound groups were also different. As mentioned in section Hydrolysate fermentation sample analysis, the EA method had reliable measurement for aromatic compounds, while the EC methods mainly detected carboxylic acids and furans. This difference in analytical method in relation to metabolomics results was also seen during statistical model building, as EA-GC-MS data-sets could predict Glu CR and EtOH PR properly, but failed to model lag-phase on its own, which was validly predicted by EC-GC-MS data. Accordingly, furans were mainly identified to prolong lag-phase, and aromatic compounds were mostly responsible for reduced growth. These results suggest that in a metabolomics study, it is important to have a wide coverage of detectable compounds, so that the chance of overlooking potential target compounds can be reduced [27,54]. And one way of achieving this is to use multiple analytical tools for measuring the same sample.

Furfural and HMF were reported as the two most important inhibitors in biomass hydrolysates, which delay as well as reduce growth [13,40,48,55]. In the growth test of this study, it was found that furfural indeed prolonged lag-phase at a concentration of 0.2 g/l, but HMF did not display any inhibitory effect until its concentration reached above 0.5 g/l (Table 5). However, when tested combined, HMF enhanced the negative effect of furfural on lag-phase, and reduced growth rate. When HMF was tested combined with other compounds, which showed no effect individually, like levulinic acid, 2-furoic acid and pantoyllacton, inhibition took place, resulting in extended lag-phase and decreased growth rate (Table 8). These observations suggest that HMF probably functions as a co-inhibitor in biomass hydrolysate, for which inhibition is mainly the result of synergistic effects. Furthermore, synergistic effect reduces the threshold concentration for inhibition. For instance, both HMF and syringaldehyde showed toxicity only at 1.0 g/l towards the fermenting yeast, but when tested combined, the inhibitory effect was present at 0.5 g/l (Table 5). So it is possible that when multiple inhibitors are present, the toxicity threshold of HMF and syringaldehyde reduce to below 0.1 g/l, which is close to their reported concentration in biomass hydrolysates [11,14,41,42].

A group of compounds that were identified with Glu CR and EtOH PR models showed no effect in the growth test. This group of compounds consist of aromatic acids (Table 5). Earlier studies demonstrated that aldehyde was the most toxic form of aromatic compounds, the corresponding acids were less, while the alcohol form was the least toxic [20,22,50]. This was confirmed in this study, and was clearly illustrated with vanillin and vanillic acid, of which the acid form had no effect, while the aldehyde form almost abolished growth at 1.0 g/l (Table 5). Besides the identification of previously reported inhibitors in biomass hydrolysates [9,15,17,50,55,56], two new compounds were found to be toxic, which are sorbic acid and phenylacetaldehyde. As shown in Table 5, both compounds already showed significant inhibitory effect on growth at 0.2 g/l. The high toxicity towards the fermenting yeast indicates that these two compounds are important inhibitors in biomass hydrolysates. Though not recorded as hydrolysate inhibitors, sorbic acid was described as a preservative weak acid, which disturbs yeast growth through uncoupling mechanism [8,55,57,58], while phenylacetaldehyde was known of having antibiotic activity in maggot therapy [59]. It should be mentioned that the enzyme cocktail used in this study also contains sorbic acid, so the sorbic acid detected in biomass hydrolysates was partially from addition of the hydrolyzing enzyme in most feedstock hydrolysates.

Of the potential inhibitory compounds identified by the statistical models, about half are unknowns. Some of these compounds are on the very top of the ranking lists, see Additional files 4 and 5. Since most of the known compounds identified by the models showed inhibitory effect towards the fermenting yeast in growth tests, it is expected that there are also important/novel inhibitors among the unknown compounds. Identification needs to be conducted for these unknown compounds to verify this, which will be the next step in identifying lignocellulosic biomass hydrolysate inhibitors.

The inhibition property of these compounds was linked to their presence in lignocellulosic biomass hydrolysates through applying metabolomics approach. To our knowledge, this is the first systematic study on identifying inhibitory compounds in lignocellulosic biomass hydrolysates using a non-targeted approach.

## Conclusion

Inhibitory compounds in lignocellulosic biomass hydrolysates were successfully identified through applying an exometabolomics approach. The identification was conducted by relating the fermentability of biomass hydrolysates with their composition using statistical models, (n)PLS-2CV. The non-sugar composition of biomass hydrolysates were analyzed with two GC-MS methods, using ethyl acetate extraction and ethyl chloroformate derivatization to remove sample sugar contents, respectively. Besides the known inhibitors, sorbic acid and phenylacetaldehyde were for the first time identified as inhibitors among the identified compounds in lignocellulosic biomass hydrolysates.

## Methods

### Biomass hydrolysate preparation and fermentation

24 different hydrolysates were prepared from six types of biomass, by using four different hydrolysate preparation methods. The six types of biomass were sugar cane bagasse (Bag) (Zillor, Brazil), corn stover (CS) (University of Cape Town, South Africa), wheat straw (WS) (Oostwaardshoeve, The Netherlands), barley straw (BS) (Oostwaardshoeve, The Netherlands), willow wood chips (Willow) (Oostwaardshoeve, The Netherlands) and oak sawdust (Oak) (wood-flooring supplier ESCO, The Netherlands). Prior to pretreatment, biomass (except oak sawdust) was ground to pieces of average length 3 mm and dried at 80°C for at least 16 hours. To prepare 1 l hydrolysate, 300 g dried biomass was used. The four hydrolysate pretreatment methods were dilute acid (2% H_2_SO_4_), mild alkaline (3% Ca(OH)_2_), alkaline/peracetic acid and concentrated acid (72% H_2_SO_4_). The biomass pretreated with the first three methods was hydrolyzed enzymatically, using Accellerase 1500 (Genencor®), while acid hydrolysis was used for biomass pretreated with concentrated acid (40% and 15% H_2_SO_4_). The detailed pretreatment and hydrolysis procedure was described in Zha et al. (2012) [33]. After hydrolysis, solid content was separated from the hydrolysate by filtration, and the filtrated hydrolysate was sterilized using filter sterilization and stored at 4°C before use.

Batch fermentations were carried out in 2 l New Brunswick fermentors, using 1 l of sterilized hydrolysate as substrate. The fermenting yeast was *Saccharomyces. cerevisiae* CEN.PK 113-7D (CBS 8340), and the inoculum was prepared in a 500 ml Erlenmeyer flask. The cells were harvested by centrifugation after incubating overnight in mineral medium (MM) [60] with 20 g/l glucose, and inoculated into fermentors with density of 0.1 g cell dry weight per 1 l hydrolysate. All fermentations were carried out at 30°C, under anaerobic conditions by sparging 0.5 l/min N_2_ continuously, and pH was set at 5 by adding 1 M H_2_SO_4_ or 2 M KOH.

For each of the 24 hydrolysates, one batch fermentation was conducted after checking its reproducibility [18]. During the whole fermentation process, CO_2_ concentration in the off-gas was monitored automatically and samples were taken at fixed time intervals. These samples were kept at 4°C and used to measure optical density at 600 nm (OD), glucose and ethanol concentration with either Cobas® Mira Plus (Roche) or Arena® 20 Analyzer (Thermo Scientific).

### Hydrolysate fermentation sample analysis

For each of the selected hydrolysate fermentations, cell free time samples were chosen for analyzing their overall compositions. Two GC-MS methods, namely ethyl acetate extraction (EA)-GC-MS and ethyl chloroformate derivatization (EC)-GC-MS, were used to analyze the fermentation samples.

For EA-GC-MS, the extraction was done by adding 550 μl ethyl acetate into 0.5 ml sample and vortex for 2 min. The mixture was centrifuged to separate the ethyl acetate fraction, of which 400 μl was transferred to a vial and dried under N_2_. The following internal standards in ethyl acetate were added to the same vial: phenylethanol-D5, cinnamic acid-D5 and hydroxybenzaldehyde-D4. The extraction and centrifugation process was repeated, and from the ethyl acetate fraction, another 400 μl was transferred to the same vial, after drying with N_2_, the following internal standards in pyridine were added: alanine-D4 and citric acid-D4. The extract was then oximized by adding 30 μl 56 mg/ml ethoxyamine · HCl, and incubating at 40°C for 90 min. Followed by adding dicyclohexylphtalate (DCHP) and difluorobiphenyl (DFB) in pyridine as injection standard, the oximized extract was silylated by adding 100 μl *N-*methyl*-N-*trimethylsilyl-trifluoroacetamide (MSTFA), and incubating at 40°C for 50 min. Measurement was carried out by 1 μl splitless injection in the PTV injector of an Agilent® 7890A GC with an Agilent® 5975C MS as detector. The analytical column used was a HP-5MS column (30 m × 0.25 mm × 0.25 μm).

For EC-GC-MS, the sample pH was brought above 10 by adding NaOH solution, followed by the addition of the following internal standards in pyridine: leucine-D3, succinic acid-D4 and cinnamic acid-D5. The injection standards, DCHP and DFB in pyridine, and 300 μl ethanol were also added to the sample. Then the ethylester formation was done by two rounds of adding 40 μl ethyl chloroformate into the sample and shaking it vigorously by hand for 15 sec. The reaction was stopped by adding 750 μl dichloromethane and 500 μl 1 M bicarbonate buffer. The formed derivates were extracted with dichloromethane, and the extraction was dried with Na_2_SO_4_. The measurement was carried out the same way as in EA-GC-MS method. The analytical column used was a DB-1 column (30 m × 0.32 mm × 1 μm).

The analysis results of EA-GC-MS and EC-GC-MS were reported separately in data-sets, with detected peaks as row and fermentation sample as column. The reported values were areas of the detected peaks after correction with internal standards.

### Statistical model building

The two statistical models used were partial least square with double cross validation (PLS-2CV) [31] and n-way PLS with double cross validation (nPLS-2CV) [38]. The 2CV version of the nPLS model was developed in house. The models were written as m-files in MATLAB environment (R2012a) with PLS toolbox 2.0 (Eigenvector).

#### PLS-2CV models

PLS-2CV is a linear regression model, which predicts the fermentation phenotypes with the GC-MS analysis results of the fermentation samples (data-sets). The PLS-2CV models were assessed by calculating the so-called Q^2^ values, which indicate the prediction ability of the data-sets for a specific phenotype [31]. The maximum value of Q^2^ is 1, representing that the model could perfectly predict the phenotypes. Generally, models with Q^2^ ≥ 0.5 were selected for analyzing the selectivity ratios (SR) assigned to each peaks in the data-sets. Similar to regression coefficient (‘reg’), SR is a measure for variable importance in discrimination models. Contrary to ‘reg’, SR is corrected for the influence of interfering compounds that are not related to the modeled response [61,62]. Peaks with the highest SR values were considered having the primary contribution to the model building. Among these peaks, the identified ones were selected as potential inhibitory compounds, and tested in Bioscreen C Analyzer for their effects on the fermenting yeast (see Section Potential inhibitory compound test).

To model lag-phase, the data-sets containing the first two fermentation samples (t1 and t2) were used. As listed in Table 3, the difference as well as the combination of t1 and t2 data-sets were used to build PLS-2CV model. EA-GC-MS and EC-GC-MS data-sets were modeled both separately and combined. Thus, for lag-phase, in total six PLS-2CV models were built (Table 3). These data-sets were preprocessed by conducting a ‘square-root’ transformation to reduce the nonsymmetrical distributions of the peak areas for all compounds, and this also homogenizes the heteroscedastic measurement error. Afterwards, an ‘auto-scaling’ was carried out to reduce the effect that compounds with large peak areas would dominate the regression models [63,64]. The phenotype values were ‘mean-centered’ before data analysis.

To model glucose consumption rate (Glu CR) and ethanol production rate (EtOH PR) (see Eq2 - 4), the data-sets of all five fermentation samples were used individually (t1 to t5, see Table 2). The data preprocessing was conducted in the same way as by lag-phase data-sets (Table 3).

#### nPLS-2CV models

N-way PLS (nPLS) handles multiway data-sets, and was used to model glucose consumption rate (Glu CR) and ethanol production rate (EtOH PR). In this study, the data-sets were three-way, the three ways were (1) fermentation batch, (2) time samples of each batch, and (3) analysis results of each sample. The analysis results of EA-GC-MS and EC-GC-MS methods were used both separately and combined. Similar to PLS-2CV model, the data-sets were arranged in two way and preprocessed by conducting ‘square-root’ and ‘auto-scaling’ before transforming to the three-way structure. The phenotype values were ‘mean-centered’ before model building. The nPLS-2CV models were assessed by calculating the Q^2^ values. In most cases, models with Q^2^ ≥ 0.5 were selected for analyzing the regression coefficient (‘reg’) of each peak in the data-sets, as SR for nPLS has not yet been developed. Peaks with highest absolute ‘reg’ values were considered having the most contribution for predicting the phenotypes. Among these peaks, the identified ones were selected as potential inhibitory compounds, and tested in Bioscreen C Analyzer for their effects on the fermenting yeast (see Section Potential inhibitory compound test).

### Potential inhibitory compound test

Solutions of potential inhibitory compounds were prepared in both MM with 20 g/l glucose and YPD (Yeast extract Peptone Dextrose) medium with concentrations of 0.2, 0.5 and 1.0 g/l. For those compounds saturated at 1.0 g/l, these saturated solutions (100%) and 2- or 5-fold dilutions resulting in 50% and 20% of the saturated concentration were used. Therefore, in these cases (shown in bold in Table 5) the exact concentrations are not known.

The prepared solutions were adjusted to pH 5.0 and used as media in the growth test of the fermenting yeast, *S. cerevisiae* CEN.PK 113-7D. The growth test was conducted in triplets in honeycomb plates, using Bioscreen C Analyzer (Labsystems OY). Testing volume was 400 μl, and testing condition was 30°C, no shaking. Growth was monitored by measuring OD 420-580 nm with a time interval of 15 min during the whole experiment. The detailed procedure of Bioscreen test is described in Zha et al. [33].

## Competing interests

The authors declare that they have no competing interests.

## Authors’ contributions

YZ designed the study, carried out the hydrolysate preparation and fermentation experiments, performed the data analysis, and wrote the manuscript. JW supervised and participated in the data analysis. BM conducted the fermentation sample GC-MS analysis. KO supervised the fermentation experiments. BN participated in hydrolysate preparation and fermentation experiments. LC supervised fermentation sample GC-MS analysis. AS supervised design of the study and the data analysis. PP coordinated the whole study, participated in and supervised the drafting of the manuscript. All authors read and approved the final manuscript.

## Supplementary Material

Additional file 1: Figure S1 Description of data:Trends comparison between growth rate calculated based on OD and glucose consumption rate. Growth rate: the slope of the linear part of the OD% curve (green); glucose consumption rate (Glu CR): the slope of the linear part of the glucose consumption curve (Eq2) (red). Click here for file

Additional file 2: Figure S2 The calculation results of the four phenotypes, sorted from the smallest to the largest. The ‘red’ bars are the selected 16 fermentations for exometabolomics analysis. Click here for file

Additional file 3 **EA-GC-MS results (sheet 1), and EC-GC-MS results (sheet 2).** Description of data: analysis results of the fermentation time-point samples with EA-GC-MS and EC-GC-MS methods. The 1^st^ column is the sample names, the 1^st^ row is the detected peaks, the data are peak areas after correction with internal standard. Click here for file

Additional file 4 **Lag-phase modeling results;** Description of data: ‘sheet 1’ provides the selectivity ratios (SR) of lag-phase model 2, 5 and 6 (see Table 3), and their sums; ‘sheet 2’ provides the ranking results of lag-phase models based on the sum SR; ‘sheet 3’ provides the selected potential inhibitory compounds and to-be-tested compounds based on the ranking results. Click here for file

Additional file 5 **Glu CR and EtOH PR modeling results;** Description of data: ‘sheet 1-3’ contain results of Glu CR models, and ‘sheet 4-6’ contain results of EtOH PR models. ‘sheet 1 and 4’ provide 1) the selectivity ratios (SR) of PLS-2CV models of t3, t4 and t5, and their sums, 2) regression coefficients (‘reg’) of nPLS-2CV model of t2-t5; ‘sheet 2 and 5’ provide the ranking results based on sum SR and ‘reg’; ‘sheet 3 and 6’ provide the selected potential inhibitory compounds based on the ranking results; ‘sheet 7’ combines the results in sheet 3 and 6, provides to-be-tested compounds. Click here for file
