# Supplementary figures and images for: Identifying inhibitory compounds in lignocellulosic biomass hydrolysates using an exometabolomics approach
Source: BMC Biotechnol. 2014 Mar 21;14:22. doi: 10.1186/1472-6750-14-22 (PMC3998114; doi:10.1186/1472-6750-14-22)

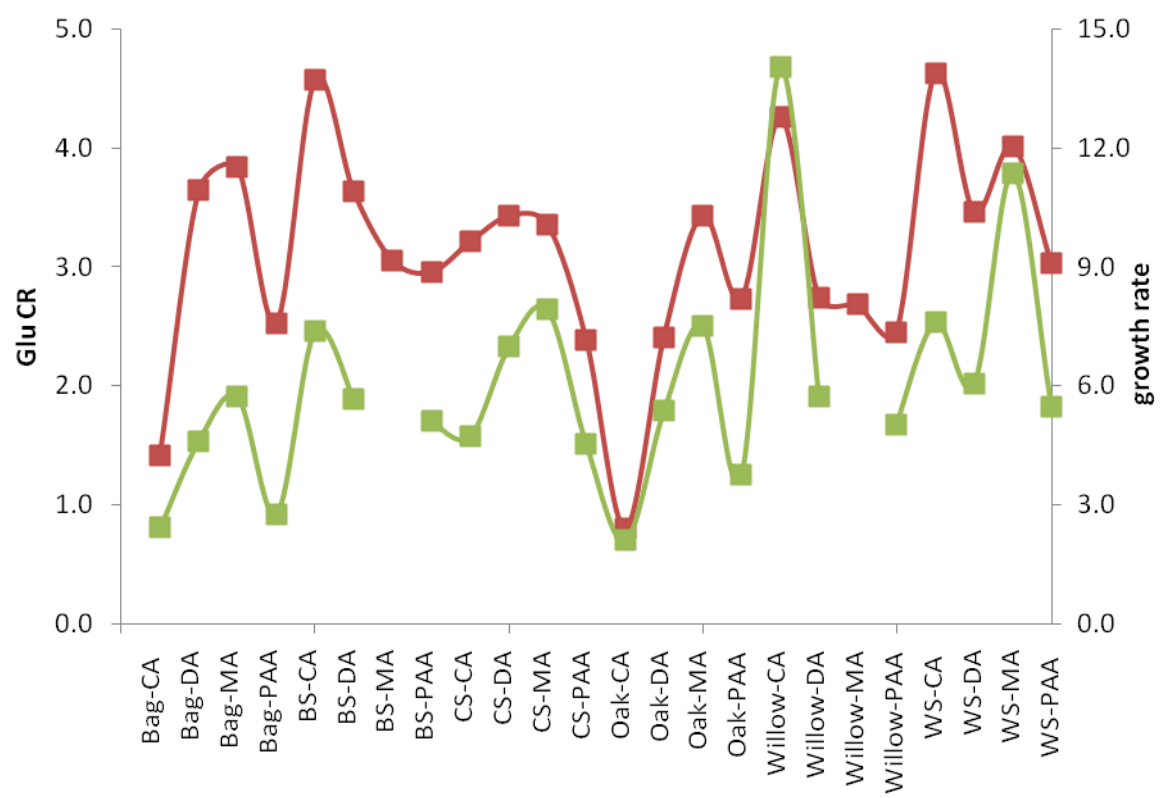

Supplement: Additional file 1: Figure S1 — Description of data:Trends comparison between growth rate calculated based on OD and glucose consumption rate. Growth rate: the slope of the linear part of the OD% curve (green); glucose consumption rate (Glu CR): the slope of the linear part of the glucose consumption curve (Eq2) (red). [file 1472-6750-14-22-S1.pdf]
