# Supplementary material for: Identifying inhibitory compounds in lignocellulosic biomass hydrolysates using an exometabolomics approach
Source: BMC Biotechnol. 2014 Mar 21;14:22. doi: 10.1186/1472-6750-14-22 (PMC3998114; doi:10.1186/1472-6750-14-22)
Supplement: Additional file 2: Figure S2 — The calculation results of the four phenotypes, sorted from the smallest to the largest. The ‘red’ bars are the selected 16 fermentations for exometabolomics analysis. [file 1472-6750-14-22-S2.pdf]

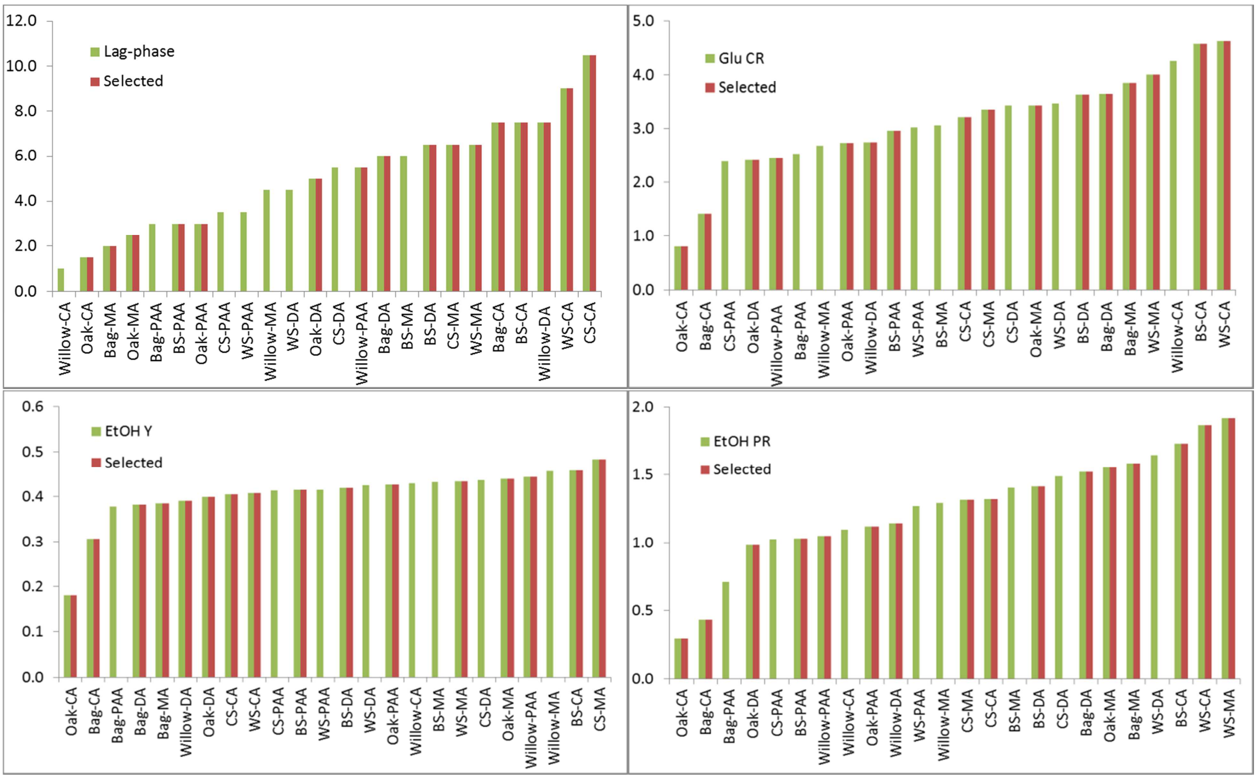
12.0
10.0
8.0
6.0
4.0
2.0
0.0
Lag-phase
Selected
Willow-CA
Oak-CA
Bag-MA
Oak-MA
Bag-PAA
BS-PAA
Oak-PAA
CS-PAA
WS-PAA
Willow-MA
WS-DA
Oak-DA
CS-DA
Willow-PAA
Bag-DA
BS-MA
BS-DA
CS-MA
WS-MA
Bag-CA
BS-CA
Willow-DA
WS-CA
CS-CA
5.0
4.0
3.0
2.0
1.0
0.0
Glu CR
Selected
Oak-CA
Bag-CA
CS-PAA
Oak-DA
Willow-PAA
Bag-PAA
Willow-MA
Oak-PAA
Willow-DA
BS-PAA
WS-PAA
BS-MA
CS-CA
CS-MA
CS-DA
Oak-MA
WS-DA
BS-DA
Bag-DA
Bag-MA
WS-MA
Willow-CA
BS-CA
WS-CA
0.6
0.5
0.4
0.3
0.2
0.1
0.0
EtOH Y
Selected
Oak-CA
Bag-CA
Bag-PAA
Bag-DA
Bag-MA
Willow-DA
Oak-DA
CS-CA
WS-CA
CS-PAA
BS-PAA
WS-PAA
BS-DA
WS-DA
Oak-PAA
Willow-CA
BS-MA
WS-MA
CS-DA
Oak-MA
Willow-PAA
Willow-MA
BS-CA
CS-MA
2.0
1.5
1.0
0.5
0.0
EtOH PR
Selected
Oak-CA
Bag-CA
Bag-PAA
Oak-DA
CS-PAA
BS-PAA
Willow-PAA
Willow-CA
Oak-PAA
Willow-DA
WS-PAA
Willow-MA
CS-MA
CS-CA
BS-MA
BS-DA
CS-DA
Bag-DA
Oak-MA
Bag-MA
WS-DA
BS-CA
WS-CA
WS-MA
